# Optimizing Productive Performance of Laying Hen and Egg Quality Under Cold Stress: A Dietary Approach With Crude Protein, Methionine and Zinc

**DOI:** 10.1002/vms3.70652

**Published:** 2025-10-21

**Authors:** Ahmad Mohebbifar, Shadi Sedgh‐Gooya, Mehran Torki

**Affiliations:** ^1^ Department of Animal Science College of Agriculture and Natural Resources Razi University Kermanshah Iran

**Keywords:** cold stress, crude protein, laying hen, methionine, productive performance, zinc

## Abstract

**Background:**

Cold stress reduces egg production and quality in laying hens by increasing energy demands for thermoregulation, thereby limiting nutrients for reproduction and disrupting ovarian function and calcium metabolism.

**Objectives:**

This study aimed to evaluate the effects of dietary crude protein (CP), methionine (Met) and zinc (Zn) levels on laying performance and egg quality of hens under cold stress conditions.

**Methods:**

A total of 432 Lohmann LSL‐Lite hens (65 weeks old) were allocated to 12 dietary treatments in a 3 × 2 × 2 factorial arrangement, with six replicates per group. Treatments included three CP levels (15.80%, 14.30%, 12.80%), two Met levels (0.3%, 0.22%) and two Zn levels (0 and 30 mg/kg). Birds were housed under cold stress conditions (5°C–13°C) for 12 weeks.

**Results:**

Reducing dietary CP improved egg production, feed conversion ratio, and egg mass (*p* < 0.05). Higher Met (0.3%) increased egg weight compared to 0.22% Met (*p* < 0.05). Significant interactions between CP and other dietary factors (Met, Zn) affected egg shape index and Haugh unit (*p* < 0.05). The lowest CP and Met levels resulted in the poorest egg shape index, while 0.22% Met combined with 14.30% CP yielded the highest Haugh units.

**Conclusion:**

Lowering CP improved laying performance under cold stress with minimal effects on most egg quality traits; however, interactions between CP and Met influenced egg shape index and albumen quality. The study concluded that optimal results were achieved with diets containing 14.30% CP, 0.30% Met and 30 mg/kg Zn.

## Graphical abstract

Higher dietary Met, increased EW. However, interactions between CP and Met levels influenced egg shape index and Haugh unit. The combination of lower Met (0.22%) and intermediate CP (14.30%) yielded the highest Haugh units, while lower CP and Met resulted in the poorest egg shape index.

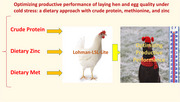

## 1 Introduction

Cold stress, defined as exposure to ambient temperatures below 16°C, is a major challenge in laying hen production (Kim et al. 2023). It reduces egg production (EP) and compromises egg quality, including egg weight (EW), shell thickness, albumen height and Haugh units, leading to substantial economic losses (Blahova et al. 2007; D. Li et al. 2020; Kim et al. 2023). These effects arise from several physiological mechanisms: energy is diverted from egg synthesis to thermogenesis, the hypothalamic–pituitary–gonadal axis is disrupted with reduced gonadotropin secretion, intestinal blood flow and nutrient absorption decline, corticosterone alters calcium metabolism and increased oxidative stress further impairs reproductive efficiency (Kim et al. 2023; D. Li et al. 2025). These challenges underscore the importance of targeted nutritional strategies to mitigate the adverse impacts of cold stress.

Crude protein (CP) is central to sustaining laying performance because it supplies the essential amino acids required for enzyme activity, tissue repair and egg synthesis (Alagawany et al. 2016). In the late laying phase, Lohmann LSL‐Lite hens (65 weeks old) typically require 14.0%–16.0% CP, depending on feed intake (FI) (Lohmann Breeders 2020). However, nutrient requirements are not static; they vary across production phases and are influenced by environmental stressors. Under heat stress (> 30°C), reduced FI necessitates higher nutrient density diets, with increased energy and amino acid concentrations but lower CP to minimize metabolic heat load (Teyssier et al. 2022). In contrast, cold stress (< 16°C) stimulates FI as hens consume more feed to maintain body temperature, thereby increasing total nutrient intake without markedly altering dietary nutrient density (D. Li et al. 2020). Despite this adaptive increase in FI, the optimal CP level for hens under cold stress remains poorly defined. Moderate CP reductions can lower nitrogen excretion and metabolic heat production, but excessive reductions compromise both performance and egg quality (Alagawany et al. 2016). Supplementation with feed‐grade amino acids, particularly methionine (Met)—the first limiting amino acid in corn–soybean meal diets—can help balance reduced‐CP diets (Zhang et al. 2024). Under normal conditions, older hens require approximately 0.35%–0.40% Met (National Research Council 1994; Zhang et al. 2024). However, under stress conditions, the demand for Met rises markedly, reflecting its essential role in antioxidant defence and methylation processes (Morales et al. 2020; Kachungwa Lugata et al. 2022).

Zinc (Zn), an essential trace mineral involved in more than 300 enzymatic processes, also plays a crucial role in supporting laying hens during stress conditions. Its physiological functions include antioxidant defence, immune regulation and maintenance of metabolic homeostasis (Prasad and Kucuk 2002). The Lohmann management guide recommends 60 mg Zn/kg diet during both rearing and laying, a value considerably higher than the National Research Council (1994) standard of 35 mg/kg, reflecting Zn's broader recognized functions. Under stress conditions, Zn requirements rise to 60–80 mg/kg due to heightened oxidative stress and reduced Zn absorption (K. Sahin, Kucuk, et al. 2002; K. Sahin et al. 2009). Numerous studies have shown that Zn supplementation enhances laying performance and egg quality by improving shell thickness, EW, Haugh unit and feed conversion ratio (FCR) (Brown and Nestor 1973; Torki et al. 2015). A recent meta‐analysis of 11 trials confirmed that dietary Zn supplementation (40–120 mg/kg) reduced FCR (−0.50 g feed/g egg) and modestly improved EP (+0.33%), EW (+0.14 g), Haugh unit (+0.64) and shell thickness (+0.84 mm) (Ogbuewu and Mbajiorgu 2022).

Although the individual effects of CP, Met and Zn on poultry under thermal stress have been investigated, little is known about their combined effects under cold exposure. Therefore, this study was conducted to evaluate the independent and interactive impacts of dietary CP, Met and Zn levels on laying performance and egg quality in hens subjected to cold stress.

## 2 Materials and methods

### 2.1 Birds, General Management and Dietary Treatments

The animal care procedures used in this study were reviewed and approved by the Ethics Committee of Razi University (Kermanshah, Iran). A total of 432 Lohmann LSL‐Lite laying hens, 65 weeks of age and with an average body weight of 1567 ± 140 g, were selected for the experiment. This age represents the late laying period, during which hens are more susceptible to environmental stressors such as cold and typically experience natural declines in productivity and egg quality (Frerichs et al. 2022).

The hens were randomly assigned to 12 dietary treatments arranged in a 3 × 2 × 2 factorial design, comprising three CP levels (15.80%, 14.30% and 12.80%), two Met levels (0.30% and 0.22%) and two Zn levels (0 and 30 mg/kg). Each treatment included six replicate groups, with six hens per replicate. Birds were housed in wire cages measuring 45 cm × 45 cm × 45 cm, with three birds per cage; two adjacent cages formed one replicate group.

All birds were maintained on a 16 h light: 8 h dark photoperiod and given 14 days to adapt to the experimental diets. The feeding trial lasted for 12 weeks, starting when the hens reached 65 weeks of age. Feed was provided at approximately 110 g per hen per day, and water was available ad libitum. Environmental conditions were monitored throughout the study, with an average indoor relative humidity of 45 ± 5%. Minimum and maximum daily ambient temperatures inside the facility ranged from 5–8 ± 1–6°C to 13–0 ± 3–1°C.

Soybean meal served as the primary protein source in the diets and was adjusted to achieve the target CP levels. DL‐Met (99% purity, Evonik Industries, Germany) was used to supplement Met levels, while Zn sulphate (98% purity, Merck, Germany) served as the Zn source. The formulation of all experimental diets followed the nutrient recommendations of the Lohmann LSL‐Lite management guide and the National Research Council (National Research Council 1994) (Table 1).

**TABLE 1:** Ingredient composition and calculated and determined chemical analyses of the experimental diets (% as‐fed basis, unless stated otherwise).

Composition (g/kg)	0.30 Dig Met^a^						0.22 Dig Met					
	Control		LCP1.5^b^		LCP3^b^		Control		LCP1.5		LCP3	
Zinc	−	+	−	+	−	+	−	+	−	+	−	+
Corn grain (78 g of CP)	62.829		65.906		68.746		62.748		66.002		68.832	
Soybean meal, 42.50 g of CP/kg	24.156		19.921		15.690		24.266		20.021		15.779	
Soybean oil	0.592		0.395		0.279		0.640		0.382		0.268	
Lime stone (CaCO_3_) (38% Ca)	9.987		9.994		10.000		9.987		9.994		10.000	
Dicalcium phosphate^c^	1.532		1.568		1.605		1.531		1.566		1.603	
Common salt	0.328		0.217		0.104		0.328		0.221		0.107	
NaHCO_3_ (Na. Bicarbonate)	—		0.163		0.332		—		0.158		0.327	
Premix for laying hens^d^	0.500		0.500		0.500		0.500		0.500		0.500	
DL‐Methionine, 990 g/kg	0.075		0.094		0.114		—		0.013		0.041	
L‐Lys‐HCl, 780 g/kg	—		0.153		0.254		—		0.122		0.251	
L‐Threonine, 985 g/kg	—		0.053		0.114		—		0.051		0.113	
L‐Tryptophan, 980 g/kg	—		0.013		0.034		—		0.012		0.034	
Filler	—		1.049		2.228		—		0.958		2.146	
Provision, % (as fed basis)												
Metabolizable energy (Kcal/kg)	2.700		2.700		2.700		2.700		2.700		2.700	
Crude protein (%)	15.800		14.30		12.80		15.800		14.30		12.80	
Calcium (%)	4.09		4.09		4.09		4.09		4.09		4.09	
Available phosphorus (%)	0.35		0.35		0.35		0.35		0.35		0.35	
Sodium (%)	0.15		0.15		0.15		0.15		0.15		0.15	
Linoleic Acid (%)	1.500		1.500		1.500		1.500		1.500		1.500	
DCAB mEq/kg^e^	171.445		171.445		171.445		171.445		171.445		171.445	
Digestible lysine	0.720		0.720		0.720		0.720		0.720		0.720	
Digestible methionine	0.300		0.300		0.300		0.220		0.220		0.220	
Digestible methionine + cystine	0.440		0.440		0.440		0.440		0.440		0.440	
Digestible threonine	0.527		0.527		0.527		0.527		0.527		0.527	
Digestible tryptophan	0.158		0.158		0.158		0.158		0.158		0.158	

^a^ Dig Met = digestible methionine.

^b^ LCP1.5 and LCP3 are low‐protein diets containing respectively 15 and 30 g less crude protein kg^−1^

^c^ Contains 18.5% P and 21% Ca.

^d^ 2400 mg; D ‐pantothenic acid, 3200 mg; niacinamide, 9600 mg; vitamin B_6_, 1120 mg; folic acid, 360 mg; vitamin B_12_, 5000 µg; vitamin C, 20,000 mg; biotin, 20 mg; vitamin K_3_, 960 mg; choline chloride 60,000 mg; 20,000 mg; copper, 1600 mg (as CuSO_4_ .5H_2_O), iron, 13,000 mg (asFeSO_4_·7H _2_ O); manganese 13,000 mg (as MnO_2_); zinc, 10,000 mg (as ZnSO_4_); cobalt, 80 mg (as CoSO_4_·7H_2_O);iodine, 200 mg (as KI); selenium, 80 mg (as Na_2_SeO_3_·5H_2_O).

^e^ DEB = dietary electrolyte balance per milliequivalent value for dietary electrolyte balance. Calculated from dietary sodium, potassium, and chloride concentration (DEB, mEq/kg of diet = Na^+^ K^+^ Cl^−^, mEq/kg of diet); calculated values.

### 2.2 Productive Performance

Data collection began after a 2‐week adaptation period to the experimental diets. Daily records were maintained for the number of eggs produced and the weight of each egg per replicate. FI, EP, egg mass (EM) and FCR were analysed on a replicate (pen) basis, with each replicate consisting of six hens housed in two cages of three hens each. EP percentage was calculated as the total number of eggs produced divided by the number of hens in each replicate, multiplied by 100. Average EW was determined by averaging the weights of all eggs collected from each replicate daily. EM was calculated by multiplying EP (%) by the corresponding EW (g). FI was measured by providing approximately 110 g of feed per hen daily and recording the leftover feed weekly; the difference was used to calculate total FI per replicate. FCR was calculated as the ratio of total FI (g) to total EM (g) for each replicate over the entire experimental period. Mortality was monitored daily and used to correct FI, EP and FCR values per replicate by adjusting for the number of live birds.

### 2.3 Egg Quality Traits

At the end of the experiment, egg quality traits were evaluated using two eggs per replicate per day over three consecutive days. The parameters assessed included Haugh unit, shape index, yolk index, shell weight, shell thickness and yolk colour. The egg shape index was calculated by measuring the egg's length and width using a precision compass (Swordfish, 0.02 mm, China), and applying the formula:

Shape Index = (Width/Length) × 100 (Lokaewmanee et al. 2023). The Haugh unit was determined using the standard formula developed by Eisen et al. (1962), based on EW and albumen height. Shell weight was determined after breaking the eggs and rinsing the shells under running water to remove residual albumen. Cleaned shells were air‐dried at room temperature for 24 h, then weighed using a digital scale with 0.01 g accuracy. Shell thickness was measured using a dial and pipe gauge (Ozaki MFG. Co., Tokyo, Japan). Measurements were taken at three specific locations on each shell: the blunt end (air cell), equator (middle) and sharp end. Before measurement, the shells were emptied, rinsed and air‐dried. The average of the three values was recorded as the shell thickness. Yolk colour was evaluated using the Roche Yolk Colour Fan (DSM, Switzerland). For the yolk index, the height (*H*) of the yolk was measured using a tripod micrometre (Mitutoyo, 0.01 mm, Japan), and its diameter (*D*) was measured with a compass (Swordfish, 0.02 mm, China). The yolk index was calculated using the formula: Yolk Index = (Yolk Height/Yolk Diameter) × 100 (Lokaewmanee et al. 2023).

### 2.4 Statistical Analysis

The GLM procedure of SAS (Statistical Analyses System 2015) was employed to analyse the data using ANOVA in a completely randomized design that had a 3 × 2 × 2 factorial arrangement of treatments. The Kolmogorov–Smirnov test was utilized to verify the normality of the data, and all statements of significance were based on a probability of less than 0.05. Duncan's Multiple‐Range test (Duncan 1955) was used to compare the mean values. The model employed for analysis was *Y*_ijkl_ = *μ + (A_i_) + (B_j_) + (C_k_) + (AB_ij_) + (AC_ik_) + (BC_jk_) + (ABC_ijk_) + (e_ijkl_)*, where *Y_ijkl_* represents the measured characteristic, *μ* is the overall mean, (*A_i_*) is the main effect of CP, (*B_j_*) is the main effect of Met, (*C_j_*) is the main effect of Zn, (*AB_ij_*) is the interaction between CP and Met, (*AC_ik_*) is the interaction between CP and Zn, (*BC_jk_*) is the interaction between Met and Zn, (*ABC_ijk_*) is the interaction between CP and Met and Zn and (*e_ijkl_*) is the residual error. In cases where the interaction was significant, the effects of the primary factors were not taken into account.

## 3 Results

### 3.1 Productive Performance

The effect of different levels of experimental treatments on productive performance under cold stress conditions is shown in Table 2. It was found that the FI was not affected by varying levels of CP, Met and Zn (*p* > 0.05). However, the different CP levels did have a significant impact on EP ratio, FCR and EM under cold stress (*p* < 0.05). Table 2 shows that the highest FCR was observed in hens fed with a diet containing normal CP, which was significantly different from the other two treatments with the lowest FCR (3.16 vs. 2.51 and 2.69) (*p* < 0.05). In addition, the highest EM and EP ratio were observed in hens fed with 14.30% and 12.80% CP compared to the control treatment (44.87 and 42.20 vs. 37.87; 71.62 and 66.89 vs. 59.58, respectively) (*p* < 0.05). The interaction effects of different experimental treatments on productive performance were not significant (*p* > 0.05).

**TABLE 2:** Effect of different levels of CP, Met and Zn on the productive performance of laying hens under cold stress conditions.

Productive performance		FI (g/hen/day)	FCR (g feed: g egg)	EM (g/hen/day)	HDEP (%)
Treatments					
CP (%)					
	NCP	110.08	3.16^a^	37.87^b^	59.58^b^
	LCP1.5	110.04	2.51^b^	44.87^a^	71.62^a^
	LCP3	110.00	2.69^b^	42.20^a^	66.89^a^
Met (%)					
	0.3	110.05	2.73	41.19	65.80
	0.22	110.02	2.84	41.19	66.25
Zn (mg/kg)					
	0	110.08	2.74	41.61	65.71
	30	110.05	2.82	41.69	66.35
*p* value					
CP		0.373	0.013	0.003	0.002
Met		0.565	0.534	0.576	0.870
Zn		0.565	0.657	0.959	0.816
CP*Met		0.717	0.636	0.489	0.600
Met*Zn		0.088	0.462	0.389	0.391
CP*Zn		0.717	0.353	0.359	0.394
CP*Met*Zn		0.373	0.866	0.600	0.678

*Note*: CP = Crude protein; Met = Methionine; Zn = Zinc; NCP = Normal crude protein; LCP1.5 and LCP3 = low‐protein diets containing respectively 15 and 30 g less crude protein/kg; FI = Feed intake; FCR = Feed conversion ratio; EM = Egg mass; HDEP = Hen day egg production.

^a–b^Means with no common superscript within each column are significantly (*p* < 0.05) different.

### 3.2 Egg Quality Traits

The effect of different levels of experimental treatments on egg quality traits is shown in Tables 3 and 4. It was found that yolk colour, yolk index, shell thickness and shell weight were not influenced by any of the treatments (*p* > 0.05). However, the level of Met had a significant effect on EW (*p* < 0.05) (Table 3). In particular, an intake of 0.3% of Met resulted in a higher EW compared to an intake of 0.22% (65.03 vs. 64.31, respectively). Furthermore, the interaction effects of Met and CP, Met and Zn, and CP and Zn on egg shape index were found to be significant (*p* < 0.05). Among the treatments tested, the lowest egg shape index was observed for the diet containing 0.22% of Met and 12.80% CP. The next lowest egg shape index was observed in the treatment containing 0.22% of Met and 14.30% CP. Both of these treatments were significantly different from the other treatments (*p* < 0.05) (Table 4). Regarding the mutual effects of Met and Zn, the treatment of 0.3% of Met and 0 and 30 mg/kg of Zn had the highest egg shape index (*p* < 0.05) (Table 4). In addition, the highest egg shape index was observed in the treatment containing normal CP without Zn and 30 mg/kg of Zn regarding the interaction between CP and Zn (*p* < 0.05) (Table 4). Finally, the mutual effects of Met and CP on the Haugh unit were found to be significant. The highest Haugh unit was observed in the treatment containing 14.30% CP and 0.22% Met, which was significantly different from the treatments containing 0.3% Met and 14.30% CP, and the treatment containing 0.22% Met and 14.30% CP (*p* < 0.05) (Table 4).

**TABLE 3:** Effect of different levels of CP, Met and Zn on the egg quality traits of laying hens under cold stress conditions.

Egg quality traits		Egg weight (g)	Egg shape index	Yolk index	Haugh unit	Yolk colour (Roch)	Shell weight (g)	Shell thickness (mm 10^−2^)
CP (%)								
	NCP	64.48	74.38^a^	38.35	78.98	6.67	5.98	38.65
	LCP1.5	64.99	63.46^b^	38.78	80.82	6.68	6.07	38.26
	LCP3	64.55	61.31^b^	39.63	80.26	6.71	5.97	38.40
Met (%)								
	0.3	65.03^a^	73.52^a^	38.60	79.56	6.63	6.00	38.78
	0.22	64.31^b^	59.24^b^	39.24	80.47	6.74	6.01	38.09
Zn (mg/kg)								
	0	65.39	66.85	38.58	80.87	6.68	6.04	38.53
	30	63.94	65.91	39.26	79.17	6.69	5.97	38.35
*p* value								
CP		0.840	0.0001	0.112	0.292	0.917	0.581	0.691
Met		0.035	0.0001	0.210	0.354	0.181	0.939	0.066
Zn		0.065	0.314	0.178	0.085	0.833	0.416	0.636
CP*Met		0.382	0.0001	0.515	0.043	0.100	0.307	0.680
Met*Zn		0.824	0.023	0.060	0.646	0.511	0.743	0.335
CP*Zn		0.951	0.040	0.721	0.796	0.919	0.957	0.799
CP*Met*Zn		0.317	0.079	0.262	0.410	0.241	0.521	0.949

*Note*: CP = Crude protein; Met = Methionine; Zn = Zinc; NCP = Normal crude protein; LCP1.5 and LCP3 = low‐protein diets containing respectively 15 and 30 g less crude protein/kg.

^a–b^Means with no common superscript within each column are significantly (*p* < 0.05) different.

**TABLE 4:** Comparison of mean interactions of CP, Met and Zn on egg shape index and Haugh unit of laying hens under cold stress conditions.

Combinations		Egg shape index	Haugh unit
Met (%)	CP (%)		
0.3	NCP	73.63^a^	79.58^ab^
0.3	LCP1.5	73.39^a^	78.61^b^
0.3	LCP3	73.55^a^	80.50^ab^
0.22	NCP	75.14^a^	78.37^b^
0.22	LCP1.5	53.53^b^	83.03^a^
0.22	LCP3	49.07^c^	80.02^ab^
Met (%)	Zn (mg/kg)		
0.3	0	72.91^a^	80.64
0.3	30	74.13^a^	78.49
0.22	0	60.79^b^	81.10
0.22	30	57.70^b^	79.85
CP (%)	Zn (mg/kg)		
NCP	0	74.10^a^	80.22
NCP	30	74.67^a^	77.74
LCP1.5	0	65.62^b^	81.71
LCP1.5	30	61.29^b^	79.93
LCP3	0	60.83^b^	80.69
LCP3	30	61.78^b^	79.83

*Note*: CP = Crude protein; Met = Methionine; Zn = Zinc; NCP = Normal crude protein; LCP1.5 and LCP3 = low‐protein diets containing respectively 15 and 30 g less crude protein/kg.

^a–c^Means with no common superscript within each column are significantly (*p* < 0.05) different.

## 4 Discussion

Cold stress imposes significant metabolic challenges on laying hens, as a large portion of dietary energy is diverted to thermoregulation rather than production (Kim et al. 2023). Birds exposed to low ambient temperatures increase FI to generate metabolic heat, which often results in greater body weight gain and poorer FCR (Kim et al. 2023). Under such conditions, dietary protein plays a dual role. While amino acid catabolism contributes to heat production, excessive CP increases the energetic costs of deamination and uric acid excretion, thereby reducing the net energy available for egg formation (Azzam et al. 2019; Kim et al. 2023).

In the present study, hens fed 14.3% and 12.8% CP diets achieved superior EP, EM and FCR compared with those fed 15.8% CP. This suggests that reduced nitrogen turnover and lower heat increment improved nutrient utilization, allowing more dietary energy to be directed toward egg formation (Ali et al. 2025). Notably, FI was not significantly affected by CP level, consistent with earlier reports (Hsu et al. 1998; Meluzzi et al. 2001; Bunchasak et al. 2005). This indicates that the performance benefits were attributable to improved nutrient partitioning rather than differences in feed consumption.

The enhanced performance observed at reduced CP levels contrasts with many earlier studies that reported improved laying performance with higher protein diets (Yakout et al. 2004; C. Novak et al. 2006; C. L. Novak et al. 2008; El‐Maksoud et al. 2011). Several factors may explain these discrepancies. Firstly, hen age is a major determinant of nutrient requirements. At 65 weeks of age, EM naturally declines and protein requirements are lower. With high FI (around 120 g/day, typical under cold conditions), white‐egg layers require only 13%–14% CP (National Research Council 1994). Thus, the 15.8% CP diet likely exceeded requirements, while 14.3% and 12.8% CP more closely matched physiological needs. Secondly, strain differences may contribute. Our study used Lohmann LSL‐Lite (white layers), whereas many earlier studies were conducted on younger brown strains. For example, Ali et al. (2025) reported that reducing CP in 43‐week‐old Hy‐Line Brown hens reduced production unless synthetic amino acids were included. Thirdly, environmental severity must be considered. The cold stress conditions in this study (5–13°C) likely intensified the negative impacts of surplus dietary protein, making moderate CP levels metabolically more efficient. These results align with the principle of precision nutrition, which emphasizes tailoring diets to physiological stage, genotype and environment to avoid the metabolic costs of nutrient excess (Moss et al. 2021). Supporting this, Awad et al. (2018) reported higher protein efficiency ratios in birds fed low‐CP diets, and Gumpha et al. (2019) found that 13% CP supported optimal production in hens aged 35–46 weeks. Conversely, some studies caution that long‐term CP reductions may limit production at later stages (Khajali et al. 2008; Alagawany et al. 2014). Overall, the evidence indicates that reduced CP can sustain or even improve performance under specific conditions, provided diets are adequately balanced for essential amino acids.

In this study, supplementation with Met and Zn did not significantly influence productive parameters. This finding is consistent with reports under thermoneutral and heat stress conditions (Karami et al. 2018; Liao et al. 2018) but differs from studies showing improved performance with Zn supplementation under cold stress (N. Sahin, Onderci, et al. 2002; Rajabi and Torki 2021). Such inconsistencies may reflect differences in Zn source, dosage, basal diet composition, hen's age or trial duration. Similarly, Met supplementation beyond requirement thresholds may not provide additional benefits. Previous studies have shown no performance improvements when Met was added to reduced‐CP diets (Rakangtong and Bunchasak 2011; Nukreaw and Bunchasak 2015), and Hsu et al. (1998) reported no benefits of supplemental Met in low‐CP diets under heat stress. Collectively, these findings suggest that once minimum Met and Zn requirements are met, further supplementation may be redundant.

### 4.1 Egg Quality Traits

Supplementing diets with a higher level of Met (0.3%) significantly improved EW compared to the lower level (0.22%). This finding aligns with previous studies demonstrating the essential role of Met, a limiting amino acid, in protein synthesis and, consequently, in enhancing egg formation and quality. The increased availability of Met likely promoted better amino acid utilization, contributing to the production of heavier eggs (Shafer et al. 1996). Our results are consistent with those reported by Harms and Russell (2003), who observed increased EW with higher dietary Met and cysteine levels. Similarly, Bunchasak and Silapasorn (2005) noted a strong positive correlation (*r* = 0.82) between Met intake and EW under tropical conditions. Cold exposure can increase fatty acid oxidation and enhance energy metabolism in chickens, creating greater demands for sulphur‐containing amino acids like Met (Kachungwa Lugata et al. 2022). This heightened requirement likely explains why the higher Met level (0.3%) was more effective in maintaining EW under the challenging thermal environment of this study (Kim et al. 2023). However, Summers et al. (1988) reported no significant effect of Met or increased CP on early egg size, suggesting that the age or physiological status of hens may influence their response to dietary amino acids. N. Sahin, Onderci, et al. (2002) also found that Zn sulphate supplementation improved EW in hens under cold stress, which contrasts with our findings, as Zn had no significant effect on EW in the current study.

Dietary treatments did not significantly affect yolk colour, yolk index, shell weight or shell thickness. This is consistent with C. Novak et al. (2006), who observed no significant effect of varying CP levels on albumen, yolk or shell percentages in laying hens aged 20–43 weeks. Similarly, Zeweil et al. (2011) reported that dietary CP levels did not markedly influence most egg quality traits. Increasing Met also had no impact on shell characteristics, aligning with Koreleski and Swiatkiewicz (2010). Likewise, Rajabi and Torki (2021) found that supplementing 40 mg/kg Zn sulphate in the diets of 65‐week‐old hens under cold stress had no significant effects on yolk colour, shell thickness, shell weight, egg index, yolk index or Haugh unit.

A noteworthy finding of the present study was the significant interaction between dietary CP and Met levels on the Haugh unit, a key indicator of albumen quality and egg freshness derived from albumen height and EW. Hens fed 14.30% CP with 0.22% Met exhibited the highest Haugh unit values, whereas either increasing Met to 0.30% at the same CP level or combining 0.22% Met with higher CP (15.80%) reduced values. These results emphasize the importance of achieving an optimal balance between CP and Met, rather than simply increasing their dietary levels. Mechanistically, albumen protein synthesis depends on adequate and balanced amino acid availability. Ovalbumin, conalbumin and other albumen proteins represent the bulk of egg white proteins, and their synthesis can be disrupted by amino acid imbalances (Z. Li et al. 2022). Kidd and Loar II (2021) reported that disturbances in amino acid balance alter the proportions of major egg white proteins, thereby reducing albumen viscosity and Haugh unit values. Similarly, Tomczak et al. (2024) demonstrated that dietary modifications can shift albumen protein content by nearly 10%, underscoring the sensitivity of albumen quality to nutritional balance. Excess Met at 0.30% may have disrupted the optimal amino acid ratio, limiting the efficient incorporation of other essential amino acids such as lysine or threonine into albumen proteins (Mousavi et al. 2013). This imbalance could have reduced albumen deposition in the magnum, leading to lower Haugh unit values despite higher Met availability.

Our findings are consistent with earlier reports showing that higher dietary CP does not necessarily improve albumen quality. Junqueira et al. (2006) and Al‐Agawany (2012) both observed no significant improvements in Haugh unit with elevated CP levels, while Gunawardana et al. (2008) similarly reported no differences across varying CP concentrations. Shim et al. (2013) proposed that although dietary protein is essential to sustain albumen production, excess CP or disproportionate amino acid supply may impair amino acid utilization and thereby reduce albumen quality. In this context, the combination of 14.30% CP with 0.22% Met likely provided a balanced amino acid profile that optimized protein efficiency and supported superior Haugh unit values in the present study.

The lowest egg shape index was observed in the group receiving 0.22% Met combined with 12.80% CP, suggesting that low levels of both may negatively affect egg shape in older hens under cold stress. Protein is vital for egg component development, including the shell and albumen. A deficiency in CP or Met may disrupt these processes, compromising egg shape. Lotfi et al. (2018) reported that higher CP levels support stronger shell development, which may help preserve egg shape. Egg shape index classifications indicate that values above 76 reflect round eggs, 72–76 indicate normal shape, and values below 72 suggest elongated eggs (Alkan and Türker 2021). Met, as the first limiting amino acid, plays a key role in amino acid balance and protein biological value (Muller and Balloun 1974; Neto et al. 2000). An inadequate Met level may disrupt this balance, impairing shell formation and leading to misshapen eggs.

The consistent observation of a lower egg shape index in all 0.22% Met treatments, regardless of Zn level, highlights the inadequacy of this Met level under cold stress conditions. It is likely that, under such stress, hens prioritized Met for critical physiological needs rather than egg formation, resulting in compromised egg shape. The highest CP level (15.80%) produced the best egg shape index, with or without Zn supplementation, emphasizing the importance of sufficient dietary protein. While Zn is known for its role in enhancing eggshell quality (Moreng et al. 1992; N. Sahin, Onderci, et al. 2002; Torki et al. 2015), Met level in this study had a more pronounced effect on egg shape index, corroborating findings by Liu et al. (2017).

## 5 Conclusions

Under cold stress conditions, dietary manipulation of CP, Met and Zn exerted both singular and combinational effects on the productive performance and egg quality of laying hens. FI was unaffected; however, reducing CP to 12.80% or 14.30% significantly improved EP, FCR and EM compared with the control. Supplementation with 0.30% Met increased EW relative to 0.22%, while interactions among CP, Met and Zn influenced egg shape index and Haugh unit. The highest egg shape index occurred with 0.30% Met combined with either no Zn or 30 mg/kg Zn, whereas the greatest Haugh unit was observed with 14.30% CP and 0.22% Met. These results indicate that targeted nutrient adjustments, rather than single‐nutrient supplementation, are critical for optimizing specific performance and egg quality traits under cold stress. A dietary framework of 14.30% CP, 0.30% Met and 30 mg/kg Zn may offer a favourable balance across multiple parameters, although no single combination maximized all traits simultaneously. Further studies are needed to refine CP requirements under varying Met and Zn levels, cold stress intensities and different age groups of laying hens.

## Author Contributions

**Ahmad Mohebbifar**: methodology, software, formal analysis. **Shadi Sedgh‐Gooya**: writing – original draft, conceptualization, investigation, validation, formal analysis. **Mehran Torki**: conceptualization, data curation, investigation, validation, supervision, resources, project administration, writing – review and editing.

## Peer Review

The peer review history for this article is available at https://www.webofscience.com/api/gateway/wos/peer‐review/10.1002/vms3.70652.

## Data Availability

The datasets generated during and/or analysed during the current study are not publicly available are available from the corresponding author on reasonable request.
